# Effect of lingual plates on generating intra-oral pressure during swallowing: an experimental study in healthy subjects

**DOI:** 10.1186/1743-0003-10-64

**Published:** 2013-07-01

**Authors:** Kazuhiro Hori, Murali Srinivasan, Cindy Barbezat, Ken-ichi Tamine, Takahiro Ono, Frauke Müller

**Affiliations:** 1 Division of Dysphagia Rehabilitation, Niigata University Graduate School of Medical and Dental Sciences, Niigata, Japan; 2 Division of Gerodontology and Removable Prosthodontics, University of Geneva, School of Dental Medicine, 19 Rue Barthelémy-Menn, Geneva 1205, Switzerland; 3 Department of Prosthodontics, Gerodontology and Oral Rehabilitation, Osaka University Graduate School of Dentistry, Suita, Japan; 4 Department of Internal Medicine, Rehabilitation and Geriatrics, University Hospitals of Geneva, Geneva, Switzerland

**Keywords:** Dysphagia, Deglutition, Tongue, Pressure, Oral, Lingual plates

## Abstract

**Background:**

Although palatal augmentation prostheses (PAPs) can improve dysphagia, their application is compromised in the absence of maxillary abutment teeth. Experimental lingual plates (ELPs) used for raising the tongue may be employed as alternative to PAPs.

**Methods:**

Influence of different ELP designs, plateau (P–type) and drop-shaped (D–type), on the intra–oral pressure during swallowing were tested. Eleven healthy dentate volunteers, with a mean age of 35.5±10.5 years, participated in this study. Tongue pressure on the hard palate was measured using an ultra-thin sensor sheet with five measuring points, whilst performing dry, 5–ml and 15–ml water swallows, with and without the ELPs *in situ*. Additional pressure sensors were installed in the lingual aspects of the ELPs, and on the vestibular aspect of the lower molars for measuring sublingual and oral vestibule pressures, respectively. Each measurement was recorded thrice. A repeated measures ANOVA was employed to verify differences in duration, maximal magnitude and integrated value for the different experimental situations. Tukey’s post hoc test was performed for comparison testing. Statistical significance was set at *p*<0.05.

**Results:**

The sequence of tongue–palate contact on the median line of the hard palate without ELPs was maintained, except for the 15 ml P–type swallow. Tongue pressure started earlier with the D–type but reached its peak nearly at the same time as without ELPs. The peak magnitude and cumulative tongue pressure against the hard palate decreased by wearing ELPs (p<0.05), but was inconsistent between the two types of ELPs and for the different swallowing volumes. Both, maximum and cumulative vestibular pressures were mostly similar or larger with P–type than that with D–type.

**Conclusion:**

D-type and P-type ELPs seem to have the inverse effect of PAPs on the palatal tongue pressure during swallowing. These first counterintuitive findings do not yet justify rejecting the basic rationale of using ELPs for the treatment of dysphagia; hence a rather biologically designed piezographic lingual plate may be more appropriate.

## Background

Dysphagia is one of the most important clinical problems encountered in the treatment, rehabilitation and care of compromised elderly. It hinders undisturbed food and liquid intake and presents an inherent risk of aspirating. In bedbound patients it is frequently associated with aspiration pneumonia, which is a possible cause of death [1-5]. The tongue and its pressure on the palate play a pertinent role in speech, deglutition, and mastication; and are of particular importance for the swallowing reflex [6-9]. Tongue pressure against the hard palate is the largest oral pressure produced during swallowing. Measurements of tongue pressure against the palate have been performed by means of sensing probes, air filled bulbs and pressure sensors on an artificial palate. Our original ultra-thin sensor sheet has enabled measurements of tongue pressure on five measuring points for the first time under nearly natural conditions and provided novel insights into the role of tongue pressure in healthy or pathological swallowing [8]. Decline of tongue pressure and unfavourable tongue-palate contact was found in acute and chronic stroke patients with dysphagia.

Common therapies for preventing aspiration and improving deglutition in dysphagic subjects include the use of palatal augmentation prostheses (PAPs), advocated with or without an inclined head posture. Head posture has been known to help in swallowing and prevent aspiration, especially the chin-down posture which greatly enhances tongue driving force of the food bolus [10]. Other benefits of head flexure during swallowing include a better laryngeal closure, descent of the epiglottis, a subtracted glosso-pharyngeal space and an increased duration of glosso-pharyngeal contact protecting airway with a more efficient bolus clearance [11-14]. Though beneficial, the oro-pharyngeal swallow with a chin-down procedure is difficult to perform and might be perceived inappropriate in a social context [10].

PAPs, in the treatment of dysphagia, function primarily as a swallowing aid [15]. The resin plates help lower the palatal contours to enhance the tongue contact and produce a more positive tongue pressure during swallowing. Depending on the patient’s disability, the resin thickness can be adequately individualized to increase the tongue pressure and improve swallowing [6]. However increasing the thickness of the resin, although beneficial in terms of increasing the tongue pressure to produce a more resourceful swallowing, may bulk the prosthesis sufficiently to compromise the retention of the appliance [16]. This problem may further be exaggerated by the absence of abutment teeth and/or unfavourable anatomical conditions like strongly atrophied alveolar ridges. Wearing the PAP is also a challenge when patients suffer from xerostomia or neurological disorders which impede muscular control of the appliance. Loss in retention of the prosthesis directly leads to discomfort and a lack of compliance. In addition, the prosthesis itself may induce inconveniences such as a gag-reflex and/or a sustaining velvo-pharyngeal insufficiency [16]. The shortcomings of the PAPs may be avoided with the use of appliances providing adequate tongue lift and pressure but without engaging the palate. However, to be efficient, these appliances must not alter the inter-occlusal freeway space, as the negative effect of increasing the vertical dimension on tongue pressure has been well investigated [17]. The use of experimental lingual plates (ELPs), as suggested in this study, would satisfactorily fulfil the above said criteria, however these have never been tested. In an earlier pilot study, it was confirmed that these lingual plates did not significantly increase the vertical dimension at rest [18].

Based on those preliminary findings we tried to investigate the influence of wearing ELPs on the biomechanics of tongue movement during swallowing firstly by analysing the sequential pattern of tongue-palate contact, and then by evaluating the change in maximal magnitude, duration and surface under the integrated signal of tongue pressure recordings.

## Methods

The ethical committee of the University of Geneva approved the study and a written informed consent was obtained from all volunteers.

### Study cohort

A convenience sample was recruited from the staff of the Geneva dental school. Eleven fully dentate volunteers (4 women, 7 men; aged 35.5±10.5 years; age range 26–60 years) took part in this experimental clinical study. A full medical and dental history was obtained from each participant; a thorough extra-oral and intra-oral examination was performed. Exclusion criteria comprised of on-going dental or orthodontic treatment, TMJ or masticatory or swallowing disturbances and neurological disorders.

### Experimental lingual plates (ELPs)

**Figure 1:**
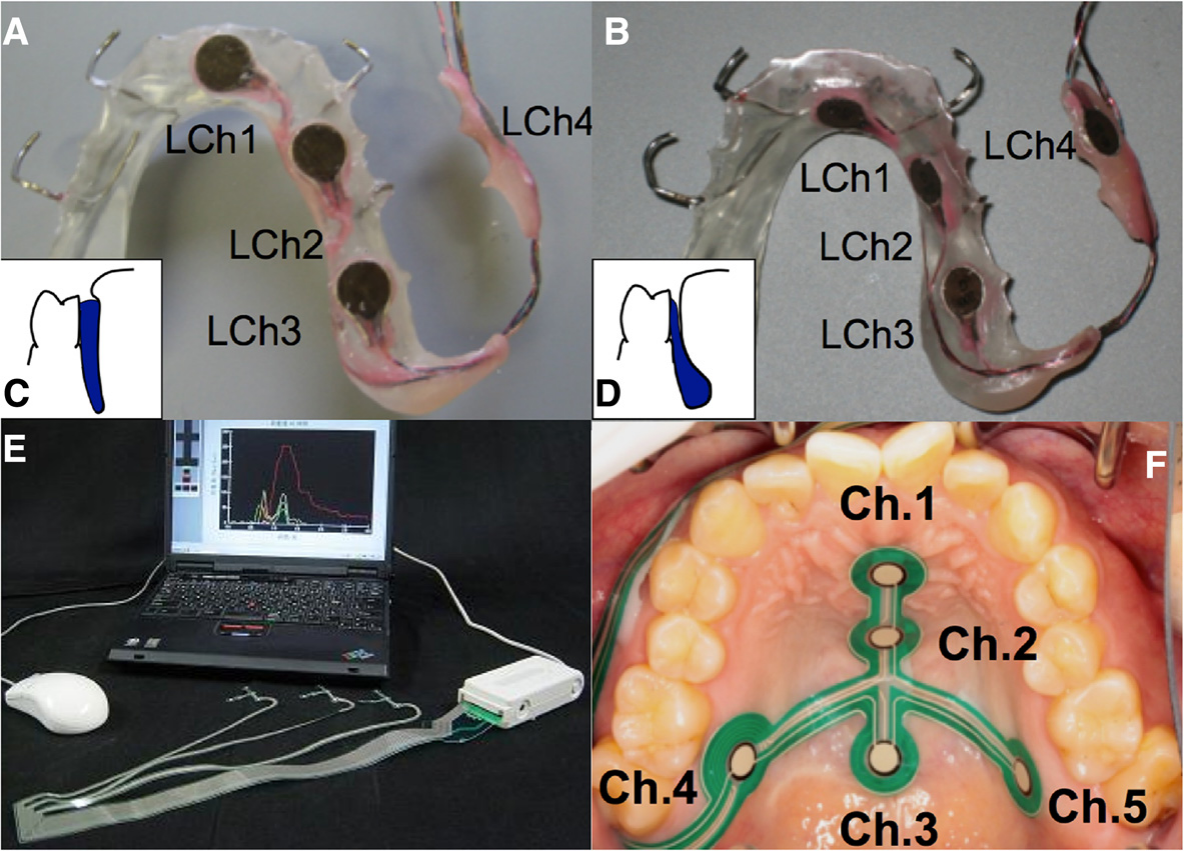
**Experimental lingual plates (ELPs). A** - P–type, and **B** - D–type. Insets (**C** and **D**) show the schematic cross-section of the ELPs. **E** - The I–scan system (Nitta, Osaka, Japan). **F** - Pressure sensors attached to the hard palate.

Two different configurations of the ELP, plateau (P–type) and drop (D–type) shapes, were fabricated using heat-polymerized polymethylmethacrylate resin. Retention was assured by bent wrought wire clasps. The P-type was shaped to extend the occlusal table lingually of the adjacent teeth (Figure 1A, C), while the D-type was inversely shaped being thin adjacent to the teeth and more voluminous sublingually (Figure 1B, D).

### Measurement of intra-oral pressure

A tactile sensor system, I-scan (Nitta, Osaka, Japan), specifically developed to measure tongue pressure on the palate was used in this study [8]. The system comprised of sensor sheets of two 0.05-mm resin film sheets. The electrodes were coated with a film of special electro-conductive ink. The electrodes, in the sensor, measured pressure by reading the changes in the electrical resistance value. The data from the electrodes in the sensor sheets were transmitted to a personal computer (PC) via a cable wire (Figure 1E). The accuracy of the sensors to tongue pressure was 0.27 kPa as reported in earlier studies [19]. The thickness of the sensor sheet was approximately 0.1 mm that adapted well to the palatal contours [7,20,21]. Five sensors in the sheet (UCh1–5) monitored tongue pressure on the palate (Figure 1F). Three sensors were aligned along the midline of the palate (UCh1 – antero-median; UCh2 – mid-median; UCh3 – postero-median), while two sensors (UCh4 & UCh5) were situated at the posterior region of the palate in the region of the greater palatine foramen. The sensors were affixed on the palate using a suitable denture adhesive (Touch Correct II, Shionogi, Osaka, Japan). The system offers a choice of 3 sizes of sensor sheets (small, medium, large), which were selected according to the size of the palate [8]. The cable wire connecting the sensors to the PC did not interfere with oral function, especially during swallowing. After attaching the sensor sheet to the palate, calibration was performed by applying negative pressure by means of a vacuum pump through an air duct within the cable of the sensor sheet [8,10]. The latter was contained in a pouch, thus the resistance could be gained by applying a certain negative pressure within the pouch. Using this technique, the calibration for all channels was obtained. The sensors were calibrated prior to the recordings, using a vacuum pump, as described in previous studies. Three additional sensors (LCh1–3) were attached unilaterally to the lingual aspects of the ELP in order to monitor the downward pressure of the tongue on the plate during the experiments. Lastly, a single sensor (LCh4) was affixed on the vestibular aspect of the lower molars to record the buccal pressure whilst performing the experimental tasks. To avoid thermal drift, an accommodation period of 15 minutes was applied before measurements calibration and measurements were performed.

### Experimental protocol

Plaster casts were fabricated from alginate impressions to fabricate the ELPs. The ELPs were verified for fit, comfort, and checked for not disturbing the occlusion.

After clinical fitting of both types of ELP, an accommodation period of 15 minutes was allotted before the calibration and measurements were performed. Participants were seated in an upright posture whilst the tongue pressures were recorded during swallowing with and without the ELPs. The swallowing tasks were performed with an upright head posture with no ELPs, followed by the P–type and D–type plates *in situ*. The participants were instructed to fix their gazes at a mark on the wall to stabilize the Frankfurt horizontal plane [10]. Three different swallowing tasks were performed for each posture, both with and without the ELPs: dry command swallow, 5 ml and 15 ml wet swallows. The temperature of the water for swallowing was maintained precisely at 37 degree Celsius to avoid thermal drift. A single operator signalled the participants before the recordings were initiated. The participants were instructed to keep the water on the tongue before swallowing. Each swallow for every experimental situation was performed thrice, so that a total of 27 recordings per subject were available for analysis.

### Data analysis

Sequential order of each pressure production (onset, peak and offset), duration, maximal magnitude and surface of the integrated signal of each sensor recording were used as outcome measures. The three repetitions per test were averaged per participant. To test the differences in the sequential order of tongue pressure production at each measuring point, the Friedman test was employed, and if significant changes were found, post hoc analyses by Wilcoxon signed-ranks tests with Bonferroni correction were performed. Differences in duration, maximal magnitude, and integrated value of tongue pressure between groups were tested using the Student t-test at a significance level of 0.05. A repeated measures ANOVA was employed to verify differences in duration, maximal magnitude and integrated value with no plates and with P–type and D–type plates, and comparison testing was performed using Tukey’s post hoc test. Statistical significance was set for a level of *p*<0.05. The statistical analysis was performed using the SPSS statistical software (SPSS Statistics, Version 20.0). A post-hoc power analysis was performed using G*Power 3.1 for sample size verification [22].

## Results

### Effect of ELPs on the sequential order of oral pressure production

**Figure 2:**
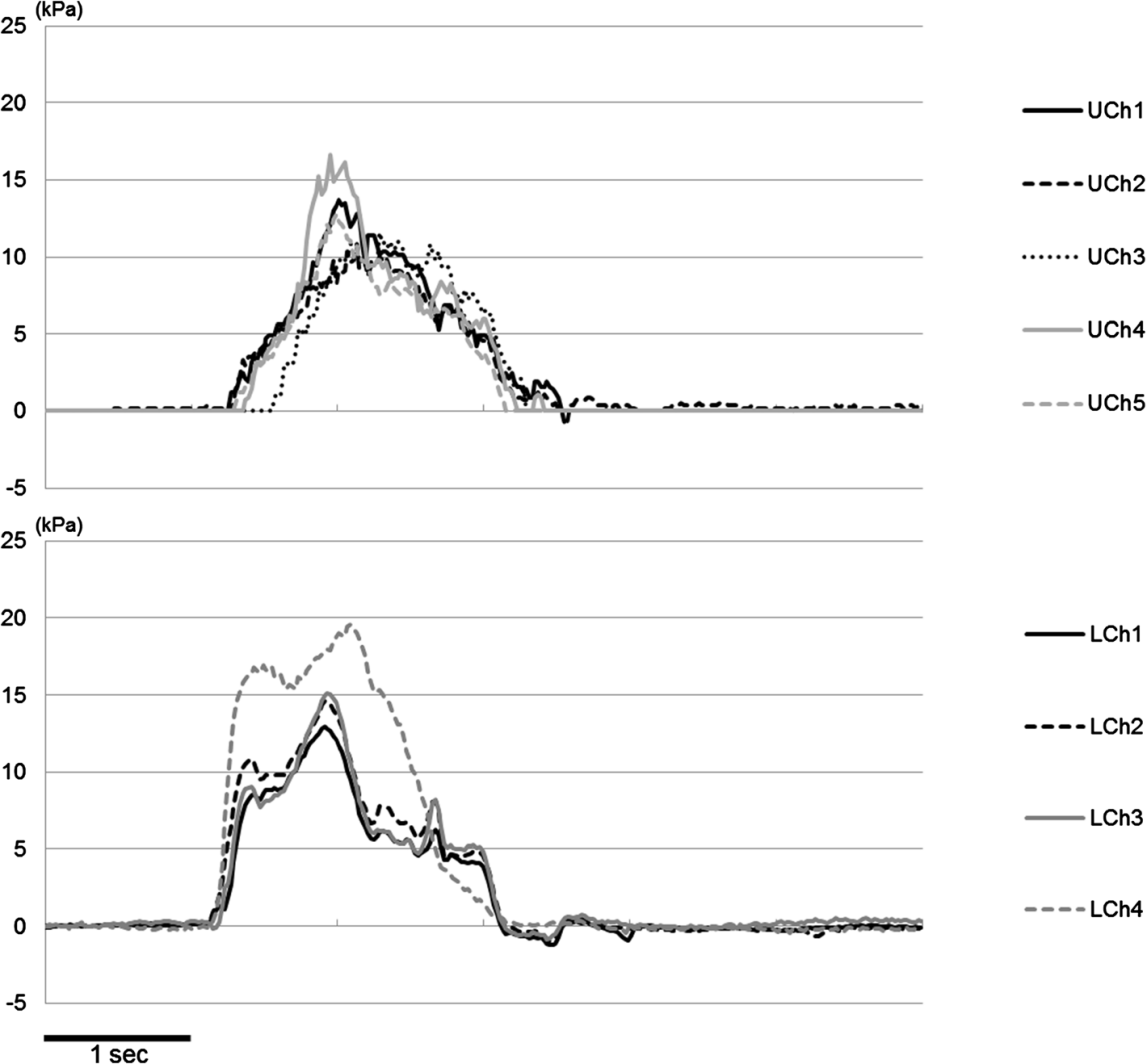
A representative wave of tongue pressure against hard palate at five measuring points (UChs1-5) and pressure on the lingual aspect of ELPs (LCh1-3) as well as oral vestibular pressure (LCh4).

**Figure 3:**
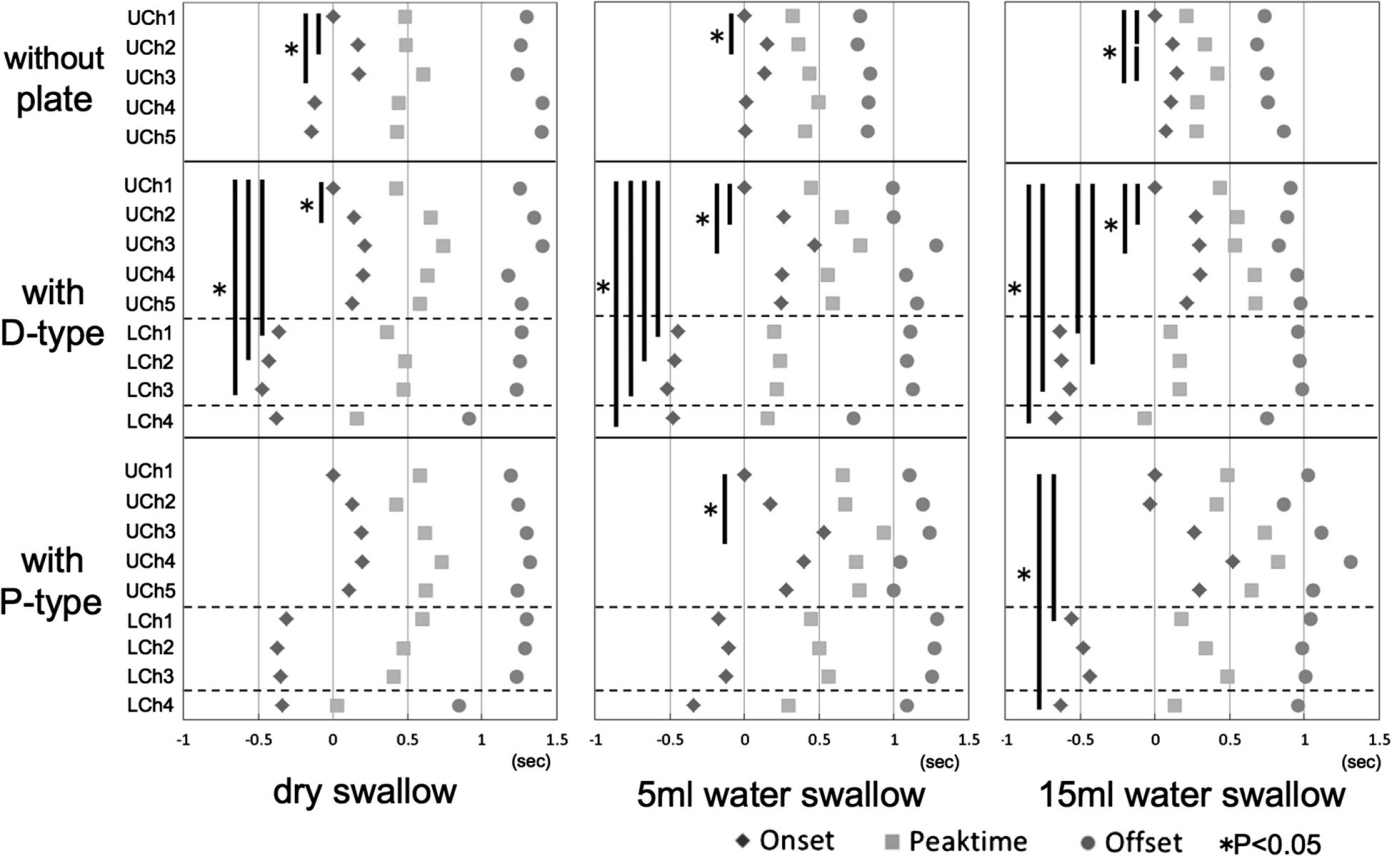
**Sequential order of tongue pressure against hard palate (UChs1-5), the lingual aspect of ELPs (LChs1-3) and the oral vestibule (LCh4) with and without ELPs during dry and 5 ml- and 15 ml-water swallow.** Onset of tongue pressure at UCh1 was set to 0s. Vertical lines with * indicate significant differences of pressure onset.

Tongue pressure traces showed a wave form against the hard palate at the five measuring points of the upper sensor sheet (UCh1–5) and on the lingual aspect of the ELP (LCh1–3) as well as the oral vestibule (LCh4) as seen in Figure 2. The sequential order of each sensor with and without ELPs during dry, 5 ml– and 15 ml– water swallows displayed differences in tongue pressure production on the hard palate and in tongue pressure on the lingual aspects of ELPs as well as the oral vestibule both, among the various bolus volumes and the ELPs *in situ* or not (Figure 3). Tongue pressure onset in UCh1 was earlier than in UCh2 and UCh3 during each bolus volume without ELPs, while peak and offset of tongue pressure did not differ. This normal sequential pattern of tongue pressure [7] remained with D–type, but disappeared in dry swallow and 15ml water swallow with P–type. LCh1–3 did not show any consistent change in tongue pressure with either type of ELP. The onset of tongue pressure production at LCh1–3 with D–type was earlier than at UCh1; but had no different onset on the hard palate than with P–type. Only LCh1 in the P–type, during 15 ml water swallow, started earlier. Timing of tongue pressure offset at LCh1–3, during each swallowing task, was similar in both types of ELPs. Onset of vestibular pressure (LCh4) with D–type was earlier than that at UCh1 during 5 ml– and 15 ml– water swallows. With the P–type onset of vestibular pressure was earlier than that of UCh1 during 15 ml water swallow. Vestibular pressure with D–type stopped earlier than palatal and lingual pressures for each bolus volume, except for UCh4 and UCh5 during 5 ml water swallow. A similar pattern was observed at the end of vestibular pressure with the P–type for dry swallow except for UCh4, UCh5 and LCh1–3, but not during the 5 ml– and 15 ml– water swallows.

### Effect of ELPs on the duration of oral pressure

**Figure 4:**
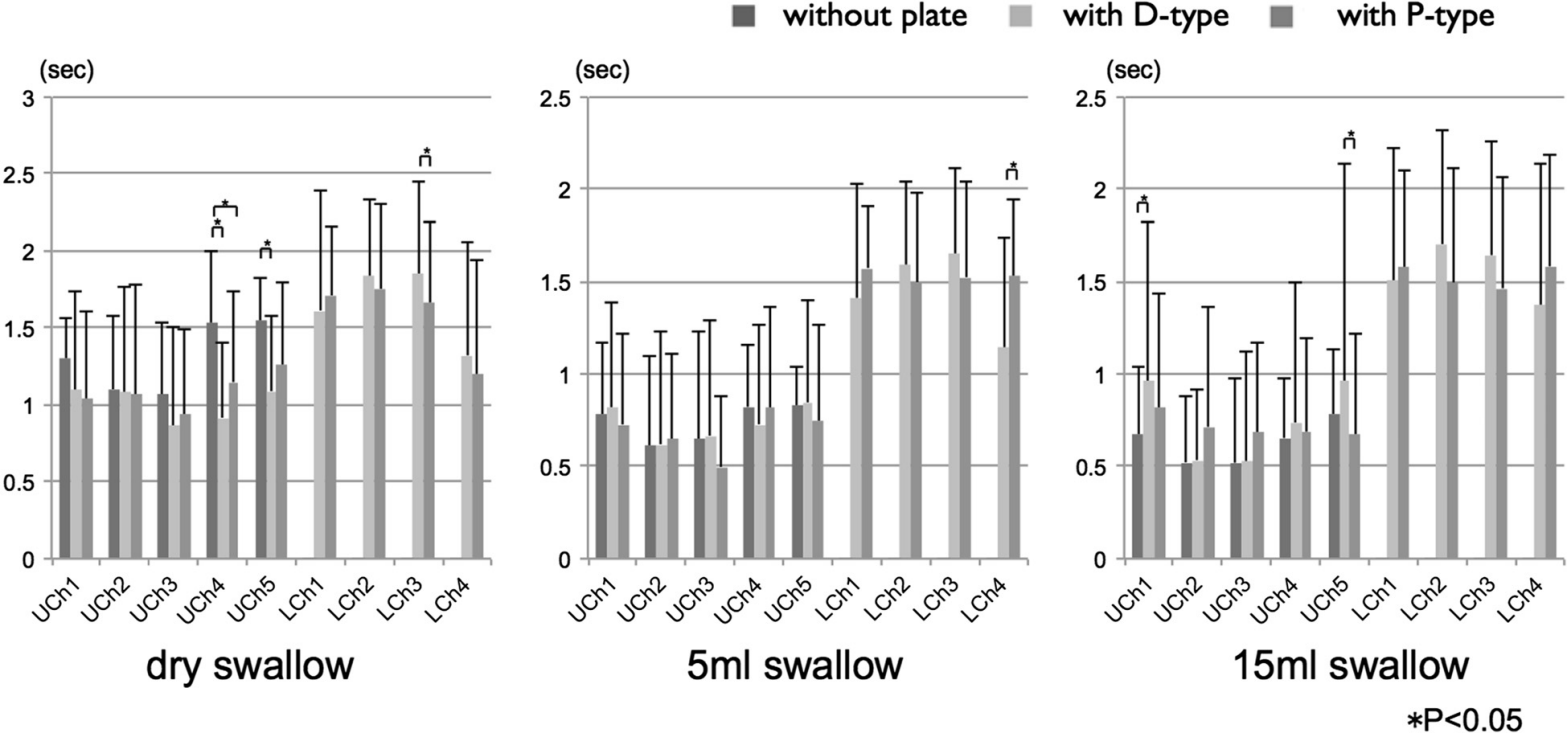
Duration of tongue pressure against hard palate (UChs1-5) and the lingual aspect of ELPs (LChs1-3) as well as the oral vestibule (LCh4) with and without ELPs during dry and 5 ml- and 15 ml-water swallow.

The duration of pressure without and with ELPs during dry, 5 ml– and 15 ml– water swallow is shown in Figure 4. Although the duration of tongue pressure on the hard palate decreased at UCh4 and UCh5 with both ELPs during dry swallow and increased at UCh1 with D–type during 15 ml water swallow, it did not change by wearing ELPs during 5ml water swallow. There was no difference in the duration of tongue pressure on LCh1–3 between ELPs during dry, 5 ml– and 15 ml– water swallows except for LCh3 during dry swallow. The vestibular pressure on LCh4 with D–type lasted shorter than that with P–type during 5 ml water swallow, but there was no difference in duration of vestibular pressure between ELPs during dry and 15 ml water swallows.

### Effect of ELPs on the maximal magnitude of oral pressure

**Figure 5:**
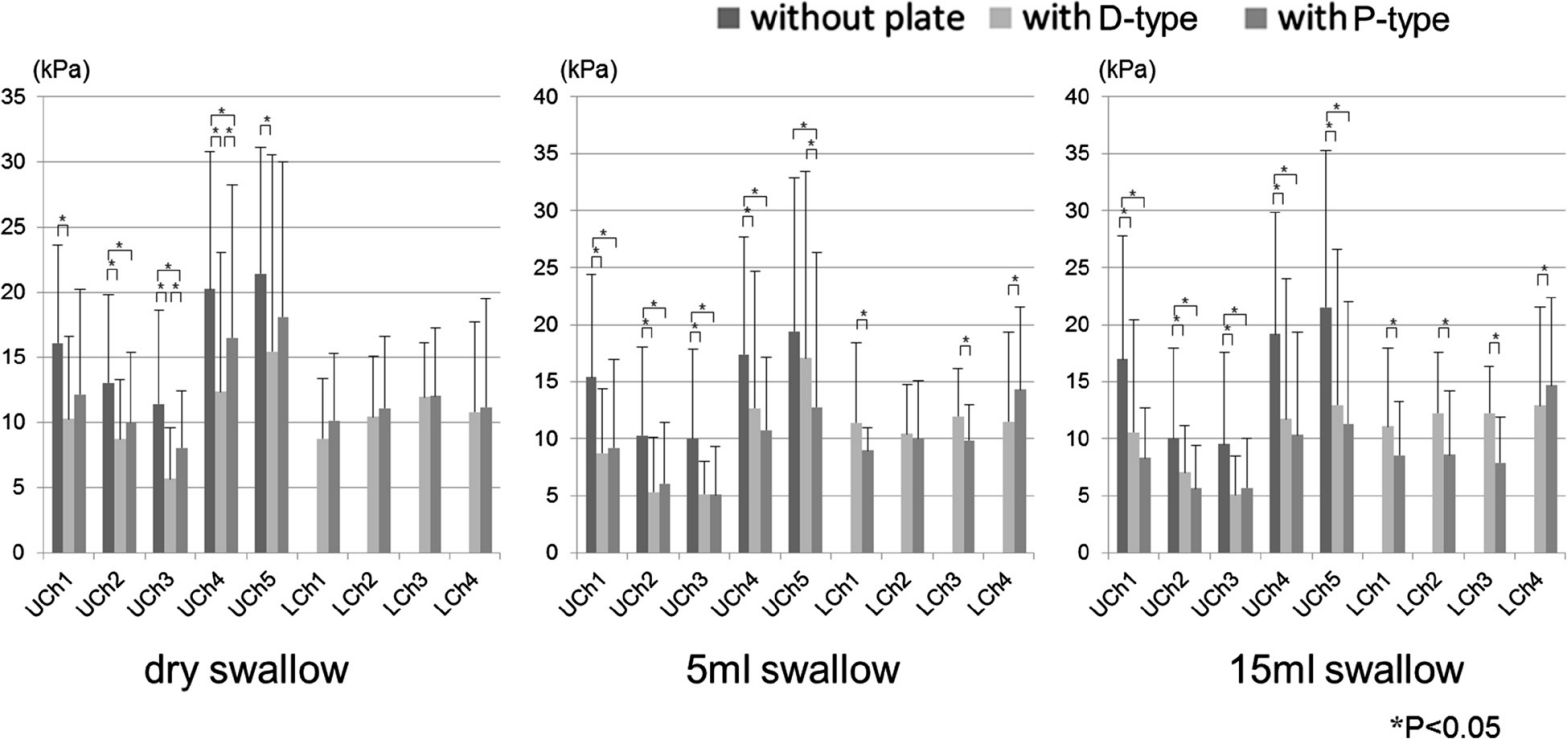
Maximal magnitude of tongue pressure against hard palate (UChs1-5) and on the lingual aspect of ELPs (LChs1-3) and the oral vestibule (LCh4) with and without ELPs during dry and 5 ml- and 15 ml-water swallow.

Maximal magnitude of pressures without and with ELPs during dry, 5 ml– and 15 ml–water swallow are shown in Figure 5. The magnitude of tongue pressure during each swallowing task decreased when wearing both ELPs in UChs1-5. The magnitude had a tendency to be larger with P–type than with D–type but was only significant for UCh3–4 (p<0.05). The maximal magnitude of tongue pressure on LCh1–3 was similar to UCh1 for each swallowing task. No significant differences in LCh1-3 were found for either ELP, but the pressure was larger with D–type than with P–type during 5 ml– and 15 ml– water swallows (p<0.05). Maximal magnitude of oral vestibule pressure in the molar part (LCh4) had no difference between ELPs during dry swallow, but was smaller with D-type than that with P-type during the 5 ml– and 15 ml– water swallows (p<0.05).

### Effect of ELPs on cumulative pressure (surface under the integrated signal)

**Figure 6:**
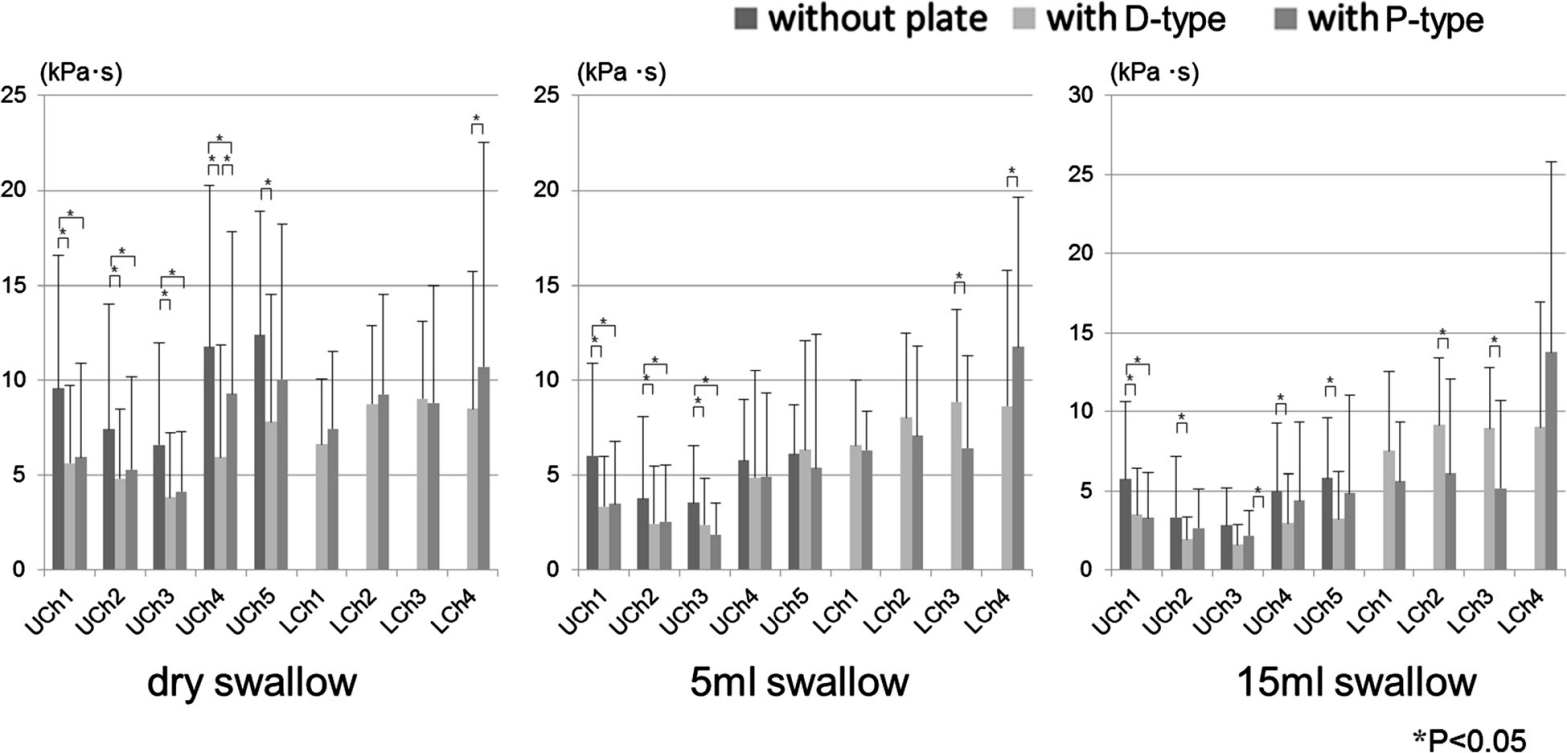
Total surface under the integrated signal of tongue pressure against hard palate (UChs1-5) and on the lingual aspect of ELPs (LChs1-3) and the oral vestibule (LCh4) with and without ELPs during dry and 5 ml- and 15 ml-water swallow.

The cumulative pressure, as in surface under the integrated signal, without and with ELPs during dry, 5 ml– and 15 ml– water swallows is shown in Figure 6. At UCh1–5 the cumulative pressure during dry swallow decreased by wearing both ELPs compared to the cumulative pressure without ELPs. Cumulative tongue pressure on the hard palate during 5ml water swallow decreased at UCh1–3 but did not change at UCh4–5 by wearing both ELPs. Cumulative tongue pressure on the hard palate during 15 ml-water swallow decreased at each measuring point when wearing D–type, but only at UCh1 for the P–type. Cumulative tongue pressure on LCh1–3 showed no difference between both ELPs during dry swallow, but was larger with D–type than with P–type at LCh3 during 5 ml water swallow and at LCh2–3 during 15 ml water swallow. Cumulative vestibular pressure at LCh4 had a tendency to be smaller with D–type than with P–type ELP.

## Discussion

The underlying principle in the management of dysphagia is to increase the tongue pressure on the palate and consequently precipitating a more forceful swallow [23,24]. This is successfully achieved by lowering the palatal contours by increasing the thickness of a palatal resin plate [6]. PAPs have been successful in treating dysphagic patients, however, they have some disadvantages [16]. The advantages of ELPs seem overwhelming, the prime advantage being the elimination of a plate on the palate, which immediately enhances patient comfort, taste sensation, speech, natural feel and finally compliance. The possible complications associated with weight-related retention of palatal appliance may also be eliminated with ELPs.

The principle of the proposed ELPs is to increase the tongue pressure on the palate by encroaching into the tongue space. This restriction would decrease the available intra-oral volume and by doing so, direct the tongue muscles towards the palate. It is hypothesised that this intended tongue lift leads to a more positive palatal contact, subsequently applying more intense tongue contact on the palate and thereby producing a resourceful swallow. The P–type plate extended a flat occlusal table lingually to the adjacent teeth. This lingual extension of the appliance was expected to force the tongue upward. The D–type plate used a more “biological” approach, which encroached into the inferior tongue space by exploiting existing space and following the natural contours of the floor of the mouth. It worked with a similar rationale but had an inverse shape.

The results of this pilot study however evidenced that the tongue pressure on the palate during swallowing did not increase as expected whilst using the proposed ELPs. This finding could be accounted for by numerous reasons. Firstly, one of the primary factors accountable could have been an insufficient bulk of the plates; in this pilot study the volume of the plates was not standardized. Studies on PAPs have indicated that the thickness of the resin plates influences the tongue pressure during swallowing [6]. Secondly, the absence of an increased pressure on the palate with the ELPs could be a subconscious reflex to their presence in the oral cavity. The subjects may have adopted an unconscious increase in freeway space at rest due to emotional factors or stress [25,26], in order to maintain the familiar “feel” of tongue contact to the anterior part of the palate at rest. However, previous experiments justify rejection of this hypothesis [18]. Furthermore, the sensor cables leaving the oral cavity and the delicate experimental set-up might have certainly raised consciousness during swallowing, as no preliminary session was used to familiarize the subject with the experimental set-up and the lingual plates. Thirdly, the shape of the plates per se or the regions of the extension of the ELPs possibly did not provide the required thrust on the tongue to influence its role in swallowing. In complete denture wearers, the retentive pressure on the lower denture applied by the tongue is higher on its anterior lingual aspects than on the posterior segments [27]. This may be attributed to the shape and the fibre orientation of the major extrinsic lingual muscle, the *genioglossus*. which has a complex range of actions [28,29]. The force of the *genioglossus* muscle on the anterior thirds of a dental arch is rather exerted downwards than laterally [27]. Hence the ELPs may have not been ideally shaped for harvesting this muscle’s potential in augmenting their function. Fourthly, another reason could have been is that the cross-sectional shapes of both ELPs were chosen arbitrarily and were not functionally generated. Tongue pressure on the hard palate revealed that the normal sequential order of tongue – palate contact on the median line was maintained with D–type but not significant with P–type. This interesting difference suggested that the shape of the P–type might interfere with the execution of forward–backward tongue movement during swallowing and this might have been less disturbed with the D–type. Piezographic techniques are well advocated in developing lingual retention in lower dentures by precluding overextensions and customizing contours [27]. The authors speculate that probably a piezographically – developed form might have a better potential in generating higher tongue pressure than the currently used forms. The injection of a standardized volume of impression material for manufacturing a piezographic plate would further diminish the variables in the experimental design. Such functionally shaped appliances would probably be a more efficient swallowing aid by less interfering with the oral functions and minimize the patient’s awareness. They may also be better tolerated in daily functions like mastication and speech. This stated hypothesis however remains to be tested. Fifthly, the action of the tongue itself may have been a causative factor for the low palatal pressure development with the present ELPs. Complete denture wearers develop muscular skills to retain their lower dentures by a downward tongue pressure up to 250N [30]. With the ELPs in situ, the tongue may have had a tendency in attempting to retain the plates in place. However, the volunteers who participated in this study were fully dentate healthy subjects who did not have any previous denture experience. The presence of abutment teeth also provided sufficient retention for the plates, hence no muscular skill was necessary to hold the plate in place. Therefore it is unlikely that they had applied such downward tongue pressure. A final train of thought that might explain the absence of an increased tongue pressure could be that the swallowing reflex is a deeply programmed pattern of muscular activity, which may take only little in consideration the initial tongue position. Thus once the process is initiated, it will continue with little reference to afferent information from the oral cavity.

An important finding in this study was the lower duration of tongue pressure on the median hard palate than the posterior–lateral parts during the dry swallow, interestingly this was not the case during the 5 ml– and 15 ml– bolus swallows. This could be attributed to the fact that the dry swallow was basically harder to perform as the bolus size was small and the viscosity of saliva was higher than that of water bolus swallows. This characteristic finding, can be assumed, was moderated by the presence of the ELPs. In addition the maximal and cumulative magnitude of tongue pressure against the hard palate was decreased at most of measuring points by wearing ELPs. This effect was most prominent in dry swallow. Conversely, a decrease in cumulative pressure was found only at the antero-median part (UCh1) during 15 ml water swallow. These results suggested that the streamlining effect on bolus driving force by ELPs was most prominent during the dry swallow and obscure in the water swallows with diffusive boluses. A significantly earlier onset of tongue pressure on the lingual aspects in comparison to the hard palate was present, although this was not significant for the dry and 5 ml swallows with the P-type. Whereas the offset of tongue pressure on both ELPs was synchronized with that on the hard palate. These findings suggest that both ELPs received tongue pressure during the whole sequence of swallowing. The drop – shape of the D–type might allow the forward movement of tongue at the beginning of swallow and then provide the support for upward movement. Such “tongue-supporting effect” by D–type is suggested by the synchronicity between the peak timing of tongue pressure on D–type and the onset of tongue pressure on the hard palate. On the other hand, the plateau – shape of the P–type might interrupt the forward movement of tongue and then result in the loss of normal sequential pattern of tongue-palate contact on the median line. The strain of cheek during swallowing interrupts the bolus entering into oral vestibule and may be intensified when the tongue action of bolus envelopment is deteriorated. There was a tendency for the maximal and cumulative magnitude of oral vestibular pressure to be smaller with D–type than with that of the P–type, suggesting the advantage of D–type. Thus, the drop – shape of D–type was a more “tongue-friendly” shape, which could allow the smooth tongue movement and deliberate a normal sequential order of tongue–palate contact with less tension from the cheeks.

Finally, as with all clinical experimental studies, methodological limitations have to be borne in mind when interpreting the results of this study. The sample size of the subjects included in this study was relatively small. However, given the high power revealed by the post-hoc power analysis it can be concluded it was large enough to avoid type II errors [22]. The only outcome measure was tongue pressure. The location and motion of the bolus were not monitored by videofluorography. Furthermore, only three volumes were tested and only liquid boluses were investigated. However, the selected volumes were found to be adequate based on previous similar experiments [7,10]. As mentioned before, the allotted adaptation time to the appliance and the research environment may have influenced the results of this study [31]. A further issue might have been that our sensor sheet system did not measure negative pressure, but in a previous study we found no such phenomenon during swallowing [7]. Even if it had occurred in the present experiments, it would not have affected the answer to the current hypothesis.

## Conclusions

The results of these clinical experiments confirm that neither D–type nor P–type ELPs are effective in increasing tongue pressure on the palate during wet or dry swallow. They seem to even have an inverse effect of PAPs on tongue pressure. However, the present pilot experiments were not designed to verify the efficiency of bolus propulsion. These first counterintuitive findings do not yet justify rejecting the basic rationale of using ELPs for the treatment of dysphagia, as a rather biologically designed piezographic lingual plate may be more appropriate.

## Abbreviations

PAP: Palatal augmentation prosthesis; ELP: Experimental lingual plate; TMJ: Temporo-mandibular joint; P-Type: Plateau-shaped experimental lingual plate; D-Type: Drop-shaped experimental lingual plate; UCh1: Palatal antero-median sensor; UCh2: Palatal mid-median sensor; UCh3: Palatal postero-median sensor; UCh4 & UCh5: Posterior palatal sensors; LCh1-3: Lingual aspect sensors on the experimental lingual plates; LCh4: Vestibular sensor on the experimental lingual plates.

## Competing interests

The authors declare that they have no competing interests.

## Authors’ contributions

KH participated in the study design, performed the experiments in Geneva, analysed and interpreted the results, performed the statistical analysis, prepared the final figures and revised the manuscript. MS was involved in drafting and revising the manuscript for important intellectual content, and in the final approval of the version to be published. CB participated in the design of the study and conducting the experiments on the subjects. KT participated in performing experiments in Geneva and contributed to the statistical analysis. TO participated in the design of the study, analysed and interpreted the results, contributed to the statistical analysis and gave important intellectual input to the manuscript as senior author. He also provided the funds for KH and KT to come to Geneva for performing the experiments. FM conceived and participated in the design of the study, participated in performing the experiments, analysed and interpreted the results and contributed to writing the manuscript in its final version as senior author. All authors read and approved the final manuscript.

## Authors’ information

K H - DDS, PhD. Associate professor of Division of Dysphagia Rehabilitation, Niigata University Graduate School of Medical and Dental Sciences, Niigata, Japan.

M S - BDS, MDS, MBA. Lecturer, and Research Assistant, Division of Gerodontology and Removable Prosthodontics, School of dental medicine, University of Geneva, Geneva, Switzerland.

C B - DMD. Division of Gerodontology and Removable Prosthodontics, School of dental medicine, University of Geneva, Geneva, Switzerland.

K T - DDS, PhD. Senior resident of dental hospital Osaka University, Suita, Japan.

T O - DDS, PhD. Associate professor of Department of Prosthodontics, Gerodontology and Oral Rehabilitation, Osaka University Graduate School of Dentistry, Suita, Japan.

F M - Dr. med. dent. habil. Professor and Head, Division of Gerodontology and Removable Prosthodontics, School of dental medicine, University of Geneva; Head of the Gerodontology Unit of the University Hospitals of Geneva, Geneva, Switzerland.

Takahiro Ono and Frauke Müller are senior authors.
